# CD44^+^/CD24^- ^breast cancer cells exhibit enhanced invasive properties: an early step necessary for metastasis

**DOI:** 10.1186/bcr1610

**Published:** 2006-10-24

**Authors:** Carol Sheridan, Hiromitsu Kishimoto, Robyn K Fuchs, Sanjana Mehrotra, Poornima Bhat-Nakshatri, Charles H Turner, Robert Goulet, Sunil Badve, Harikrishna Nakshatri

**Affiliations:** 1 Department of Surgery, Indiana University School of Medicine, Indianapolis, IN 46202, USA; 2 Department of Anatomy and Cell Biology, Indiana University School of Medicine, Indianapolis, IN 46202, USA; 3 Department of Pathology, Indiana University School of Medicine, Indianapolis, IN 46202, USA; 4 Walther Oncology Center, Indiana University School of Medicine, Indianapolis, IN 46202, USA; 5 Walther Cancer Institute, Indianapolis, IN 46208, USA; 6 Orthopedics Research Labs, Indiana University School of Medicine, Indianapolis, IN 46202, USA; 7 Department Biochemistry and Molecular Biology, Indiana University School of Medicine, Indianapolis, IN 46202, USA

## Abstract

**Introduction:**

A subpopulation (CD44^+^/CD24^-^) of breast cancer cells has been reported to have stem/progenitor cell properties. The aim of this study was to investigate whether this subpopulation of cancer cells has the unique ability to invade, home, and proliferate at sites of metastasis.

**Methods:**

CD44 and CD24 expression was determined by flow cytometry. Northern blotting was used to determine the expression of proinvasive and 'bone and lung metastasis signature' genes. A matrigel invasion assay and intracardiac inoculation into nude mice were used to evaluate invasion, and homing and proliferation at sites of metastasis, respectively.

**Results:**

Five among 13 breast cancer cell lines examined (MDA-MB-231, MDA-MB-436, Hs578T, SUM1315, and HBL-100) contained a higher percentage (>30%) of CD44^+^/CD24^- ^cells. Cell lines with high CD44^+^/CD24^- ^cell numbers express basal/mesenchymal or myoepithelial but not luminal markers. Expression levels of proinvasive genes (IL-1α, IL-6, IL-8, and urokinase plasminogen activator [UPA]) were higher in cell lines with a significant CD44^+^/CD24^- ^population than in other cell lines. Among the CD44^+^/CD24^-^-positive cell lines, MDA-MB-231 has the unique property of expressing a broad range of genes that favor bone and lung metastasis. Consistent with previous studies in nude mice, cell lines with CD44^+^/CD24^- ^subpopulation were more invasive than other cell lines. However, only a subset of CD44^+^/CD24^-^-positive cell lines was able to home and proliferate in lungs.

**Conclusion:**

Breast cancer cells with CD44^+^/CD24^- ^subpopulation express higher levels of proinvasive genes and have highly invasive properties. However, this phenotype is not sufficient to predict capacity for pulmonary metastasis.

## Introduction

Stem cell theory proposes that cancers arise from malignant transformation of normal stem/progenitor cells [1-4]. The inherent properties of stem/progenitor cells may impart their transformed counterparts with the ability to evade traditional antitumor therapies and to establish metastasis [3-5]. Tumorigenic stem/progenitor cells have been documented in hematologic malignancies as well as in solid tumors [6-8]. Several studies implicated a subset of human breast cancer cells as having enhanced ability to form tumors in immunocompromised mice [9,10]. This subpopulation of cells also demonstrated a capacity for self-renewal and generation of heterogeneous progeny. These cells were distinguished from their nontumorigenic counterparts by a specific cell surface marker profile: the CD44^+^/CD24^-^/Lineage^-^. However, the potential of these tumorigenic stem/progenitor cells to establish metastasis is not known.

Metastasis is a complex process that involves not only invasion but also homing and proliferation at sites of metastasis [11]. This process requires the activity of several genes, including the urokinase plasminogen activator (UPA)/UPA receptor system, cytokines (IL-1, IL-6, IL-8, IL-11, tumor necrosis factor, and transforming growth factor-β_1_), chemokines and their receptors (stromal cell-derived factor-1α and CXC chemokine receptor [CXCR]4), and matrix metalloproteinases (MMPs) [12-14]. Recent microarray analyses of clonal variants of MDA-MB-231 cells that home and grow at metastatic sites such as bone and lung have revealed a distinct gene expression pattern associated with metastasis to these sites [14,15]. Bone metastasis signature genes include CXCR4, connective tissue-derived factor (CTGF), IL-11, the metalloproteinase-disintegrin family member ADAMTS1 (a disintegrin and metalloproteinase with thrombospondin-1) and MMP1, whereas genes of the lung metastasis signature include CXCR4, MMP1 and cyclo-oxygenase-2. A recent study described the role of the receptor activator of nuclear factor-κB (RANK)/RANK ligand in metastasis of breast cancer cells to bone [16].

Recent gene expression profiling has challenged the long held view that metastatic cells are rare and arise during later stages of tumor progression as a result of progressive accumulation of mutations. An alternative possibility is that most primary tumors contain a subset of cancer cells that has a 'metastatic phenotype' [17-19]. We investigated whether breast cancer cells with the CD44^+^/CD24^- ^phenotype possess three essential characteristics of cells with metastatic phenotype: expression of invasion/metastasis-associated genes; invasion; and homing and proliferation at sites of metastasis. We show that a subgroup of breast cancer cell lines with a higher percentage of CD44^+^/CD24^- ^cells express higher levels of proinvasive genes and invade matrigel *in vitro*. However, the CD44^+^/CD24^- ^phenotype is not sufficient for homing and proliferation at sites of metastasis.

## Materials and methods

### Breast cancer cell lines

Except as indicated, breast cancer cell lines were obtained from American Type Tissue Culture Collection (Manassas, VA, USA). SUM1315 cells were obtained from Asterand (Detroit, MI, USA) and maintained in Dulbecco's modified eagle medium (DMEM)/F12 (CellGro, Herndon, VA, USA) containing 5% fetal calf serum (FCS), and supplemented with 10 μg/ml insulin and 20 ng/ml epidermal growth factor. MCF-7, T47-D, MDA-MB-231, MDA-MB-436, MDA-MB-468, SK-BR-3, Hs578T and HBL-100 cells were grown in minimum essential medium containing 10% FCS. ZR-75-1 and DU4475 cells were grown in RPMI containing 10% FCS. MCF-10A cells were grown in DMEM/F12 media containing 5% horse serum and the following supplements: 10 μg/ml insulin, 20 ng/ml epidermal growth factor, 100 ng/ml cholera euterotoxin, 0.5 μg/ml hydrocortisone and 2 mmol/l L-glutamine. BT474 cells were grown in RPMI containing 10% FCS, 2 mmol/l L-glutamine, 10 mmol/l HEPES, 1 mmol/l sodium pyrophosphate, 4.5 g/ml glucose, 1.5 g/ml sodium carbonate, and 10 μg/ml insulin.

### Flow cytometry

Cells were washed once with phosphate-buffered saline (PBS) and then harvested with 0.05% trypsin/0.025% EDTA. Detached cells were washed with PBS containing 1% FCS and 1% penicillin/streptomycin (wash buffer), and resuspended in the wash buffer (10^6 ^cells/100 μl). Combinations of fluorochrome-conjugated monoclonal antibodies obtained from BD Biosciences (San Diego, CA, USA) against human CD44 (FITC; cat. #555478) and CD24 (PE; cat. #555428) or their respective isotype controls were added to the cell suspension at concentrations recommended by the manufacturer and incubated at 4°C in the dark for 30 to 40 min. The labeled cells were washed in the wash buffer, then fixed in PBS containing 1% paraformaldehyde, and then analyzed on a FACSVantage (BD Biosciences).

### Northern analysis and reverse transcriptase polymerase chain reaction

RNA was isolated using RNAeasy kit (Qiagen, Valencia, CA, USA) and 5 μg RNA was subjected to Northern analysis, as described previously [20]. RT-PCR for IL-8 was performed using single step RT-PCR kit from Invitrogen (Carlsbad, CA, USA) and the following primers: 5'-GTA AAC ATG ACT TCC AAG CTG-3' and TTT TAT GAA TTC TCA GCC CTC-3'.

### Animal studies

The Institutional Animal Care and Use Committee approved all animal studies. For intracardiac injection studies, 10^5 ^cells were suspended in 100 μl HEPES-buffered saline and slowly injected into the left cardiac ventricle of 7-week-old female nu/nu mice using a 27-gauge needle (7 to 12 animals per cell type), as described previously [21]. Animals injected with TMD-231, LMD-231, or MDA-MB-468 cells were killed when they developed one or more of the following signs: hind limb paralysis from suspected spine metastasis, excessive weight loss, visible tumors, or labored breathing from lung metastasis. Lungs and bilateral axillary contents were collected in formalin for hematoxylin and eosin slide preparation. Animals injected with TMD-436 cells or DU4475 cells were killed 10 weeks after intracardiac injection. Lungs from animals injected with MDA-MB-468 cells (two animals) were digested with collagenase and hyaluronidase, and cancer cells were sorted by flow cytometry using PE-conjugated CD326 (EpCAM, #130-091-253; Miltenyi Biotech, Auburn, CA, USA) for enriching cancer cells. CD326-positive cells were evaluated for CD44 and CD24 status immediately after sorting or 1 week after sorting.

### Matrigel invasion assay

The invasion assay was performed as recommended by the manufacturer of the invasion assay kit (#ECM554; Chemicon International, Temecula, CA, USA). Briefly, cells were serum starved for 24 hours and 50,000 cells were placed on the top insert with matrigel. Serum-free media or media with 10% serum were placed in the bottom well. The number of cells that invaded through the matrigel was calculated using fluorometric assay. For each cell line, the fluorometric reading from bottom wells with the serum-free media was defined as one unit to determine relative change in invasion in the presence of 10% serum. Each experiment contained one or two wells without serum and two wells with serum for each cell line. The experiment was repeated twice for most cell lines except for MCF-7, which was done thrice.

### Statistical analysis

Data were analyzed with GraphPad software [22] to determine *P *values using Fisher's exact test.

## Results

### Human breast cancer cell lines differ quantitatively in the proportion of CD44^+^/CD24^- ^cells

**Figure 1:**
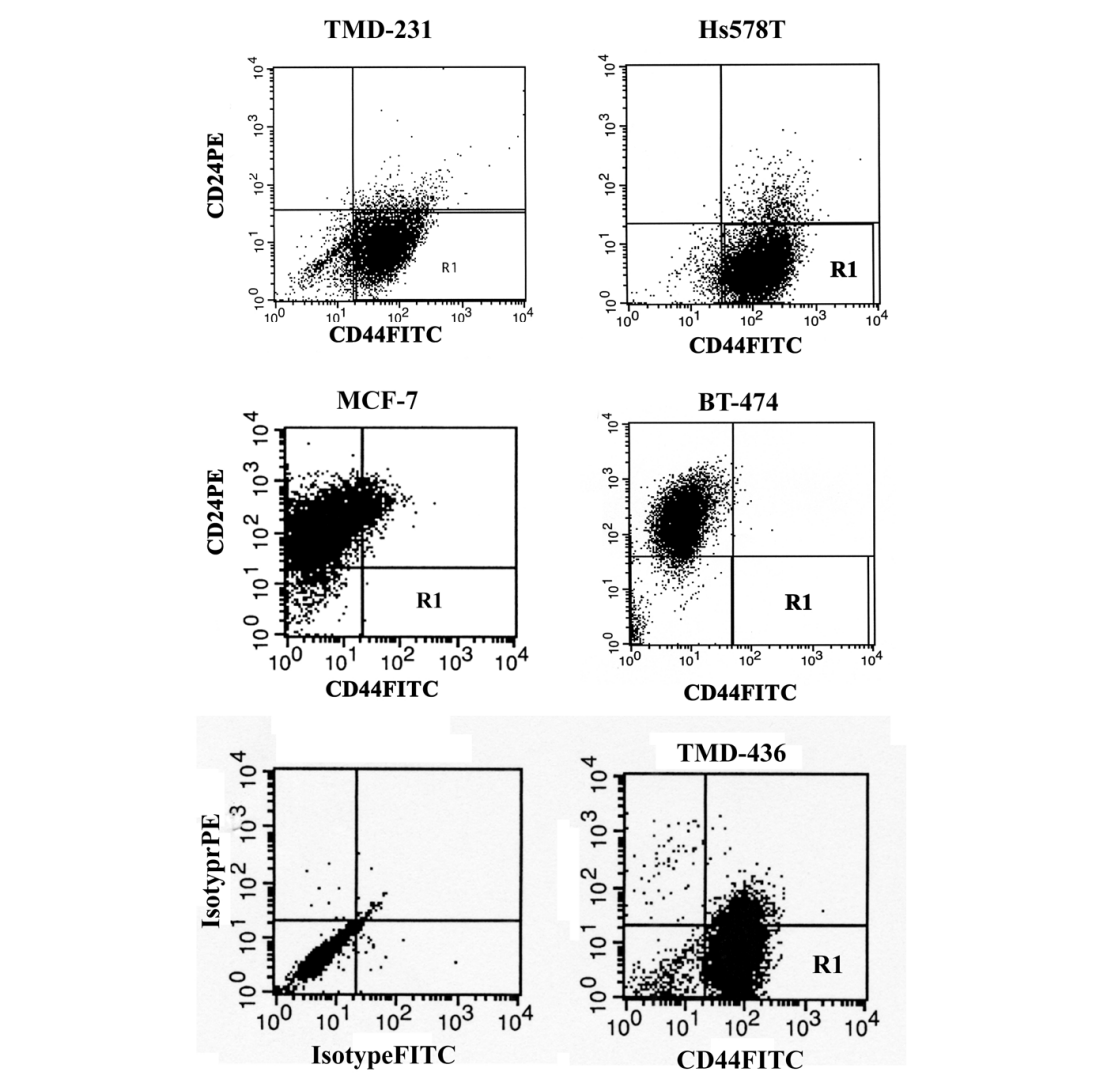
Identification of a CD44^+^/CD24^- ^subpopulation in breast cancer cell lines by flow cytometry. Cells in R1 correspond to CD44^+^/CD24^- ^cells. An isotype control corresponding to TMD-436 cells is shown.

To test the hypothesis that human breast cancer cell lines differ in the proportion of CD44^+^/CD24^- ^cells, we characterized 13 breast cancer cell lines by flow cytometry for surface expression of CD44 and CD24. In addition to parental cell lines, derivatives of MDA-MB-231 and MDA-MB-436 cells were analyzed. TMD-231 cells were derived from MDA-MB-231 cells grown in the mammary fat pad of nude mice. LMD-231 cells were derived from MDA-MB-231 cells that had metastasized to lung from the mammary fat pad of nude mice [23]. TMD-436 cells were derived from MDA-MB-436 cells grown in the mammary fat pad of nude mice. A representative flow cytometry analysis of TMD-231, TMD-436, MCF-7, Hs578t, and BT474 cell lines showing expression patterns of CD44 and CD24 is shown in Figure 1. Isotype control for TMD-436 is shown, although isotype controls were conducted for all cell lines investigated. In Table 1 the results of this analysis are summarized with respect to four fractions defined by these two markers: CD44^+^/CD24^-^, CD44^-^/CD24^+^, CD44^+^/CD24^+^, and CD44^-^/CD24^-^. This table also shows the tumor type and tissue source, cell of origin, and molecular classification based on gene expression profiling or expression of markers [24-27]. According to this classification, CK19 is expressed predominantly in cell lines of luminal type whereas CK5, CD10, and ETS1 are expressed in cell lines of basal origin. Vimentin is expressed in cell lines of mesenchymal type. MDA-MB-231 and its derived cell lines TMD-436, Hs578T, SUM1315, and HBL-100 all possessed an increased CD44^+^/CD24^- ^subpopulation (>30%). The immortalized human breast epithelial cell line MCF10A also contained a CD44^+^/CD24^- ^subpopulation. However, the percentage of the CD44^+^/CD24^- ^subpopulation in this cell line varied markedly from experiment to experiment, and appears to be dependent on the serum used for cell culture. This was not the case with transformed cell lines. Also, note that the parental and tumor-derived variants of MDA-MB-231 and MDA-MB-436 had similar CD44^+^/CD24^- ^subpopulations.

### Breast cancer cell lines with significant CD44^+^/CD24^- ^subpopulation express higher levels of genes associated with invasion

**Figure 2:**
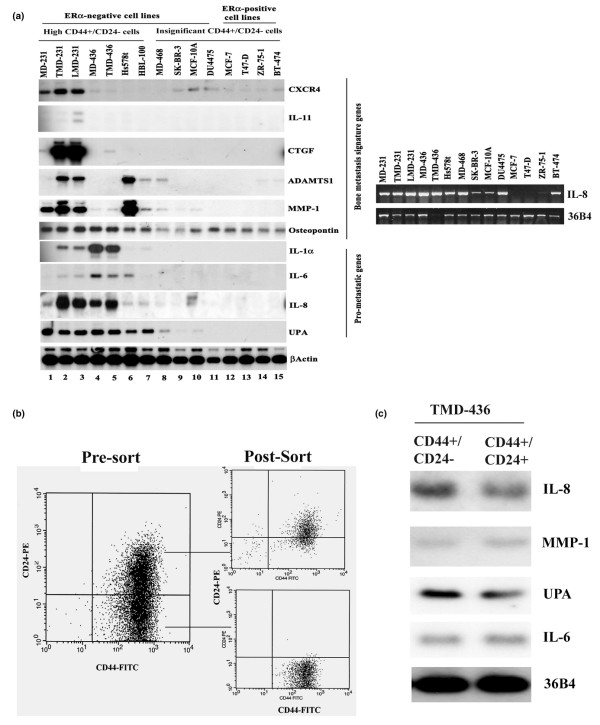
Proinvasive and 'bone metastasis signature' gene expression patterns in cell lines with variable CD44^+^/CD24^- ^subpopulation. **(a) **A representative Northern analysis of bone metastasis signature genes and proinvasive genes is shown. CD44^+^/CD24^- ^status and ER-α expression pattern in cell lines are indicated. RT-PCR results of IL-8 and the control 36B4 are shown on the right. **(b) **CD44 and CD24 expression patterns in CD44^+^/CD24^- ^and CD44^+^/CD24^+ ^subpopulations of TMD-436 sorted by flow cytometry. Expression in pre-sorted and post-sorted cells is shown. **(c) **Expression of IL-8, IL-6, MMP-1, and UPA in CD44^+^/CD24^- ^and CD44^+^/CD24^+ ^subpopulations of TMD-436 cells, as determined by Northern blot analysis. ER, estrogen receptor; MMP, matrix metalloproteinase; UPA, urokinase plasminogen activator.

To investigate the relationship between the CD44^+^/CD24^- ^phenotype and proinvasive gene expression, Northern blot and/or RT-PCR analyses were performed. Genes in a recently described 'bone metastasis signature' (CXCR4, IL-11, CTGF, osteopontin, MMP-1, and ADAMTS1) and additional genes implicated in invasion/metastasis (IL-1α, IL-6, IL-8, and UPA) were included in this analysis [12,14,15,21]. The relationship between the size of the CD44^+^/CD24^- ^population and expression of these genes is summarized in Figure 2a. Notably, only the MDA-MB-231 derivatives expressed all of the genes in the bone metastasis signature, although expression of IL-11 and CTGF was low or undetectable in the parental cell line compared with TMD-231 or LMD-231 cells. Although several cell lines expressed low levels of CXCR4, MDA-MB-231 and its derivatives exhibited the highest expression. This result was independently confirmed by flow cytometry. TMD-231 and LMD-231 cells expressed higher levels of CXCR4 (50% to 70%) than did any other cell line (data not shown). Twenty-five per cent of DU4475 cells expressed CXCR4. Of MCF-7 cells 5% to 8% expressed CXCR4 only when maintained in phenol-red free-charcoal dextran treated media, whereas the rest of the cell lines did not exhibit cell surface CXCR4. MMP-1 expression was highest in Hs578T cells and the MDA-MB-231 line and its derivatives. All other estrogen receptor (ER)-α-negative but not ER-α-positive breast cancer cell lines expressed lower but detectable levels of MMP-1. All cell lines expressed similar levels of osteopontin, suggesting that its expression is not linked to the CD44^+^/CD24^- ^phenotype.

Expression of additional proinvasive genes tested was observed predominantly in cell lines with an increased CD44^+^/CD24^- ^subpopulation. For example, expression of IL-1α, IL-6, and IL-8 was restricted to the cell lines MDA-MB-231, TMD-231, LMD-231, MDA-MB-436, TMD-436, Hs578T, and HBL-100; however, sensitive RT-PCR assay revealed IL-8 expression in several cell lines at variable levels (Figure 2a). Interestingly, tumor-derived or metastasis-derived variants of MDA-MB-231 cells demonstrated enhanced expression of these genes as compared with parental cells; this finding suggests that cells expressing these genes at higher levels have enhanced tumorigenic potential. Although present in cell lines lacking CD44^+^/CD24^- ^subpopulation, UPA expression was highest in cell lines with a higher fraction of the CD44^+^/CD24^- ^subpopulation. Consistent with microarray analysis of primary breast cancers, overall expression of proinvasive genes was higher in ER-α-negative cell lines than in ER-α-positive cell lines [28,29].

We then examined whether CD44^+^/CD24^- ^and non-CD44^+^/CD24^- ^subpopulations of cells from a single cell line exhibit differential expression of proinvasive genes. Repeated attempts to culture CD44^-^/CD24^- ^subpopulation from TMD-231, which represents under 15% of cells, were not successful because of poor plating efficiency. Therefore, we isolated CD44^+^/CD24^- ^and CD44^+^/CD24^+ ^cells from TMD-436 cells by flow cytometry (Figure 2b) and analyzed proinvasive gene expression either immediately by RT-PCR (data not shown) or after culturing for a week by Northern analysis (Figure 2c). CD44^+^/CD24^- ^and CD44^+^/CD24^+ ^cells exhibited a modest difference in IL-8 but not MMP-1, IL-6, and UPA expression (Figure 2c). These results suggest that CD44^+^/CD24^+ ^cells retain expression of the proinvasive genes that we tested.

### Invasive property is restricted to breast cancer cell lines with CD44^+^/CD24^- ^subpopulation

**Figure 3:**
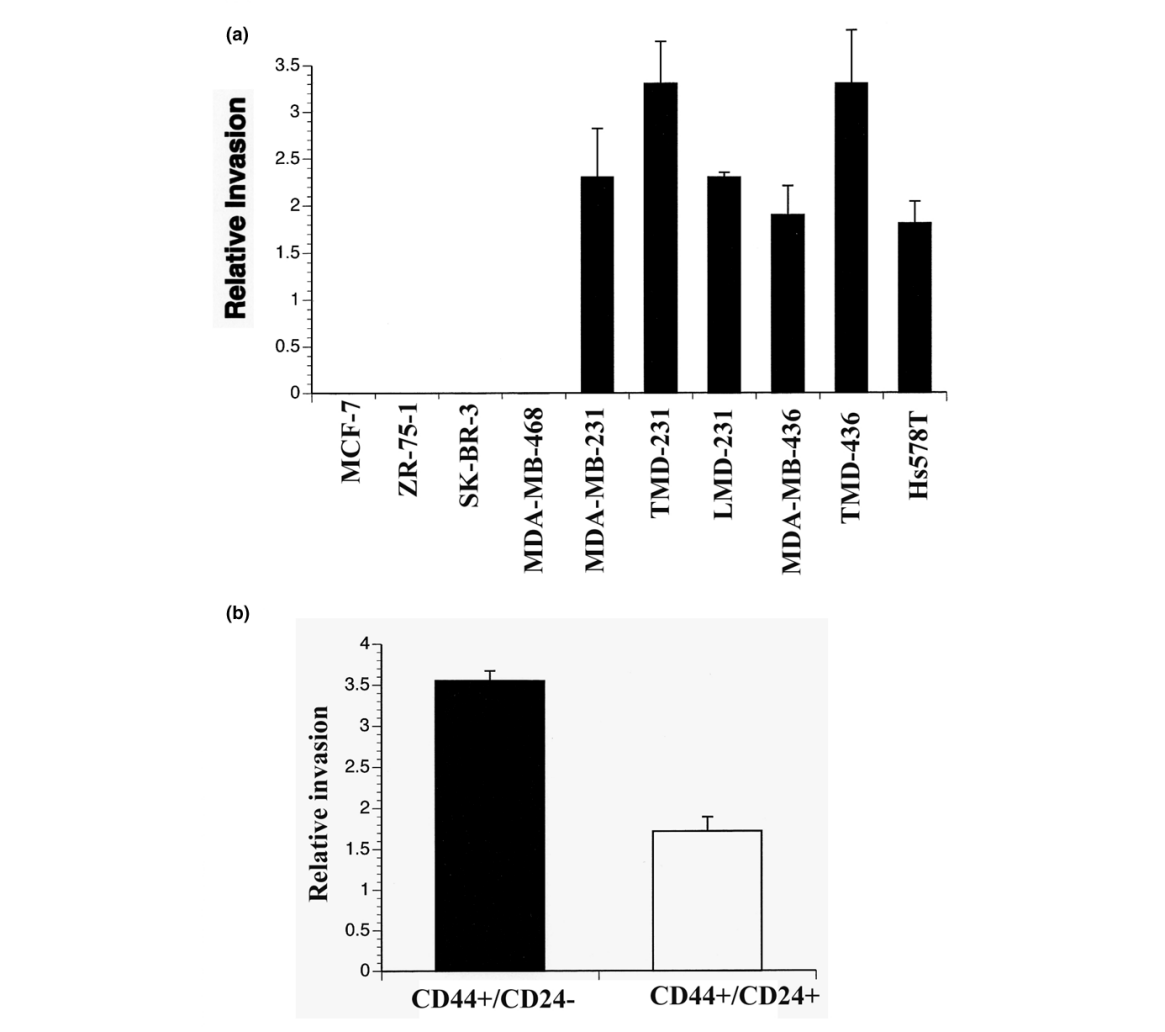
Cell lines with CD44^+^/CD24^- ^subpopulation are highly invasive. **(a) **Matrigel invasion properties of breast cancer cell lines with or without CD44^+^/CD24^- ^subpopulation. Number of cells invaded through matrigel to serum-free media for each cell line was set as one unit and relative invasion to media containing 10% fetal calf serum is shown. Mean and standard error of the mean is shown. Although invasion by TMD-231 and TMD-436 cells were higher than that of MDA-MB-231 and MDA-MB-436 cells, respectively, differences were not statistically significant. **(b) **Matrigel invasive properties of CD44^+^/CD24^- ^and CD44^+^/CD24^+ ^subpopulations of TMD-436 cells. *P *= 0.006.

Because only cell lines with a substantial fraction of CD44^+^/CD24^- ^cells consistently expressed proinvasive genes, we compared the invasive capacity of cell lines with or without CD44^+^/CD24^- ^cells. We excluded the SUM1315 line from this assay because these cells require serum and epidermal growth factor for survival, and cannot be maintained in serum-free media for 24 hours. Whereas breast cancer cell lines without CD44^+^/CD24^- ^cells lacked invasive capacity, those with CD44^+^/CD24^- ^subpopulation (MDA-MB-231, MDA-MB-436, and Hs578T) exhibited invasion (Figure 3a). There was trend toward increased invasion by TMD-231 and TMD-436 cells compared with the respective parental cells (MDA-MB-231 and MDA-MB-436), although differences were not statistically significant. These results show that the CD44^+^/CD24^- ^phenotype of breast cancer cells is associated with invasive capacity.

We also examined the invasive capacity of the CD44^+^/CD24^- ^and CD44^+^/CD24^+ ^subpopulations of TMD-436 cells. Interestingly, CD44^+^/CD24^- ^cells were more invasive than were CD44^+^/CD24^+ ^cells (Figure 3b). These results further support the association between CD44^+^/CD24^- ^phenotype and invasion.

### The CD44^+^/CD24^- ^phenotype is not sufficient to establish pulmonary metastasis

**Figure 4:**
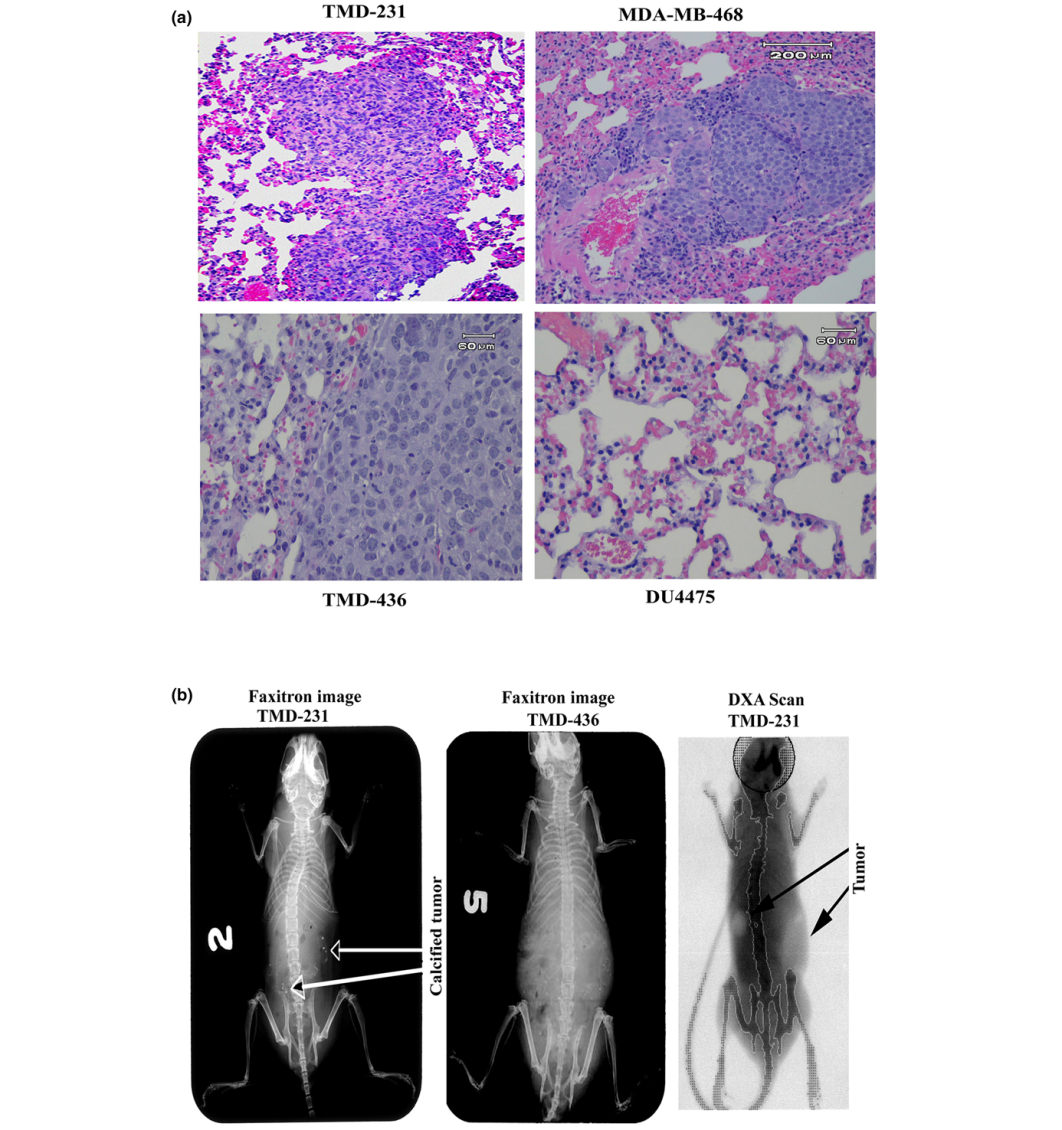
Lung metastatic properties of cell lines with or without CD44^+^/CD24^- ^subpopulation. **(a) **Hematoxylin and eosin staining of lungs in animals injected with TMD-231, TMD-436, MDA-MB-468, and DU4475 cells. Metastasis was not observed with DU4475. **(b) **Faxitron images of calcified tumor growth in animals injected with TMD-231 but not with TMD-436 cells (left and center). Dual energy X-ray absorptiometry whole-body scan of an animal injected with TMD-231 cells is shown on right.

To test the hypothesis that CD44^+^/CD24^- ^phenotype is sufficient for the establishment of metastasis *in vivo*, we tested the ability of these cell lines to establish pulmonary metastasis after intracardiac injection. In addition to the requirement of invasive property, metastatic cells should have the following properties: survival in circulation, adherence to target organ, extravasation, and initiation of growth at the metastatic site. Intracardiac injection of cancer cells into nude mice allows assessment of the metastatic capacity of cancer cells subsequent to entry into circulation [14,15,21,30-33]. Nude mice injected with cells were monitored for visible symptoms of metastasis for up to 10 weeks. The metastasis pattern of each of the cell lines tested is summarized in Table 2. TMD-231 injected animals exhibited signs of metastasis as early as 6 weeks, with most animals affected by 10 weeks. Metastatic growth was detected in vertebrae, limbs, chest wall, sternum, jaw, and scapula. Histologic staining further revealed metastatic growths in lungs (Figure 4a). Bone and visceral organ metastasis was further confirmed by dual energy X-ray absorptiometry scans and high-resolution Faxitron images (Figure 4b) and necropsy. Note that animals injected with either parental MDA-MB-231 or LMD-231 cells exhibited a similar pattern of metastasis (data not shown). Although without bone metastasis, animals injected with MDA-MB-468 cells had severe morbidity and mortality. In contrast, animals injected with parental MDA-MB-436, TMD-436, Hs578T, SUM1315, or DU4475 cells did not show any symptoms of metastasis even 10 weeks after intracardiac injection. A representative Faxitron image of an animal injected with TMD-436 cells is shown in Figure 4b. Lungs of animals injected with MDA-MB-468 or TMD-436 (6/8), but not Hs578T, SUM1315, or DU4475 cells, showed extensive growth of cancer cells (Table 2 and Figure 4a). Thus, the CD44^+^/CD24^- ^phenotype, while associated with invasion, is not sufficient for establishment of metastasis.

### Pulmonary metastasis of MDA-MB-468 cells is independent of expression of 'lung metastasis signature' genes

**Figure 5:**
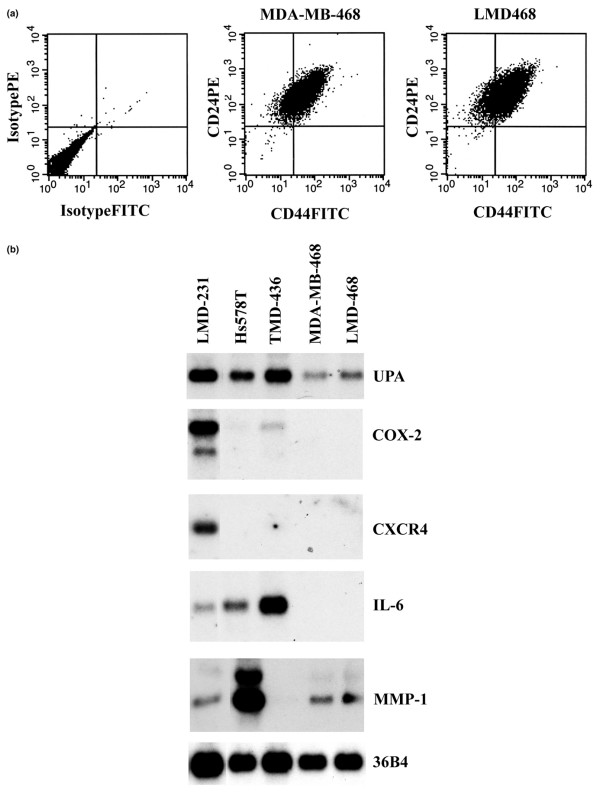
CD44 and CD24 status and lung metastasis signature gene expression in MDA-MB-468 and LMD-468 cells. **(a) **Flow cytometry showing CD44 and CD24 expression in MDA-MB-468 and LMD-468 cells. **(b) **Expression of select lung metastasis signature genes was determined by Northern blot analysis. COX, cyclo-oxygenase; CXCR, CXC chemokine receptor; MMP, matrix metalloproteinase; UPA, urokinase plasminogen activator.

CD44^+^/CD24^-^/Lin^- ^cells from primary breast cancers progress to CD44^+^/CD24^+^, CD44^-^/CD24^+^, and CD44^-^/CD24^- ^phenotypes when implanted into mammary fat pad of nonobese diabetic/severe combined immunodeficiency mice [9]. However, whether CD44^+^/CD24^- ^cells similarly progress to a heterogeneous population at sites of metastasis is not known. This possibility was explored using MDA-MB-231 (with 85% CD44^+^/CD24^- ^subpopulation) and MDA-MB-468 cells (with <3% CD44^+^/CD24^- ^subpopulation). Cancer cells that metastasized to lungs were sorted and identified using CD326 antibody, which recognizes only human epithelial cells. CD44 and CD24 expression status in parental and lung metastasized cells (LMD-231 and LMD-468) were analyzed by flow cytometry. The CD44 and CD24 expression profiles of LMD-468 and LMD-231 cells were similar to those of the parental cells (Figure 5a and data not shown). Thus, at sites of metastasis, cancer cell phenotype based on CD44 and CD24 expression does not change significantly compared with that of parental cancer cells. Also, it is unlikely that few CD44^+^/CD24^- ^cells of MDA-MB-468 cells contributed to lung metastasis and then progressed to become CD44^+^/CD24^+^.

We then examined whether LMD-468 cells express higher levels of lung metastasis signature genes than do parental cells. Lung metastasis signature genes were defined using clonal variants of MDA-MB-231 cells that grew in lungs after intracardiac injection [30]. All previously described lung metastasis signature genes that we tested were expressed in LMD-231 cells. In contrast, most of these genes were not expressed in both MDA-MB-468 and LMD-468 cells (Figure 5b). Also, TMD-436 cells, which grow in lungs, did not express the majority of the lung metastasis signature genes. These results suggest that the lung metastasis signature gene expression is not absolutely required for lung metastasis of breast cancer cells.

## Discussion

### CD44^+^/CD24^- ^phenotype of breast cancer cells is associated with invasive properties

We investigated the importance of the stem/progenitor phenotype defined by CD44 positivity and CD24 negativity for breast cancer cells to invade and metastasize. Metastasis is a complex process that involves integrated activity of genes, which function in discrete steps that include the following: angiogenesis, invasion, intravasation, survival in circulation, extravasation, and homing and proliferation at sites of metastasis [19,34]. These genes include UPA/UPA receptor, MMPs, cytokines such as IL-1, IL-6, IL-8 and IL-11, parathyroid hormone-related peptide and the chemokine receptor CXCR4 [12,14,21,31]. Here we show that several of these genes are expressed in cell lines that contain significant numbers of CD44^+^/CD24^- ^cells and that the expression pattern of these genes and the CD44^+^/CD24^- ^phenotype correlates with invasive behavior of cell lines. However, the CD44^+^/CD24^- ^phenotype is not sufficient for homing and growth at sites of metastasis. Thus, steps in the cascade of events required for the spread of cancer are dependent on distinct groups of genes, and the CD44^+^/CD24^- ^phenotype may define the expression of the group of genes involved in invasion.

CD44 and CD24 have been shown to regulate invasion and metastasis of breast cancer cells either positively or negatively. Although most studies have shown CD44-mediated invasion of breast cancer cells [35,36], Lopez and coworkers [37] showed inhibition of breast cancer metastasis by this molecule. Similarly, CD24 has been shown to promote [38] or inhibit [39] invasion and metastasis of breast cancer cells. However, association of CD44^+^/CD24^- ^phenotype with invasion observed in this study is not linked to the function of these genes because MDA-MB-468 cells expressing both CD44 and CD24 failed to invade. Also, the CD44^+^/CD24^- ^subpopulation of TMD-436 was more invasive than the CD44^+^/CD24^+ ^subpopulation of the same cell line (Figure 3b). Thus, the invasive property is intrinsic to cells of CD44^+^/CD24^- ^phenotype. Which among the genes expressed in CD44^+^/CD24^- ^cells confers the invasive phenotype is yet to be determined because CD44^+^/CD24^- ^and CD44^+^/CD24^+ ^subpopulations of TMD-436 cells exhibited modest differences in expression levels of the proinvasive genes tested but exhibited differences in invasion. In the original study on tumorigenic breast cancer progenitor cells [9] CD10 and CD140b were used as lineage markers, and so cancer cells expressing CD10 or CD140b were excluded from progenitor cells. However, several breast cancer cell lines that we examined express CD10 and it is considered a basal cell marker [25]. Studies are underway to determine whether the CD44^+^/CD24^- ^subpopulation can be further subdivided based on the expression of markers such as CD10 and whether such subsets have unique invasion/metastasis properties.

The invasive metastasis properties of several of the cell lines that we used in this study were examined by others before the identification of CD44^+^/CD24^- ^cells, and most of these studies correlated invasive properties with ER-α status and/or expression status of mesenchymal markers such as vimentin or MMPs [24,40,41]. These studies established a general trend toward increased invasiveness of ER-α-negative breast cancer cells. However, not all ER-α-negative cells were invasive (MDA-MB-468 and SK-BR-3 cells, for example) [24,42]. Our study clearly shows a direct association between the CD44^+^/CD24^- ^phenotype and invasion. However, neither our study nor previous studies revealed an association between homing and proliferation at sites of metastasis and ER-α status, mesenchymal marker expression, or CD44^+^/CD24^- ^phenotype. For example, the ER-α-positive and vimentin-negative cell line MCF-7 lacking CD44^+^/CD24^- ^subpopulation forms osteosclerotic bone lesions on intracardiac injection in nude mice [32]. Similarly, we observed lung metastasis of MDA-MB-468 cells, which are ER-α negative and vimentin negative, and lack a CD44^+^/CD24^- ^subpopulation. In contrast, the vimentin-positive and ER-α-negative cell line Hs578T, which has an 86% CD44^+^/CD24^- ^subpopulation, failed to form lung metastasis. The minor difference in our results and previously published data with respect to hematogenous metastasis of Hs578T on mammary fat pad injection [24] is probably due to low frequency of metastasis (10%). Also, we did not observe metastasis of SUM1315 cells, with a 97% CD44^+^/CD24^- ^subpopulation, in nude mice, although previous studies have shown bone metastasis of these cells in nonobese diabetic/severe combined immunodeficiency mice with humanized but not mouse bone [43].

In a complementary study, Abraham and coworkers [44] reported that the prevalence of CD44^+^/CD24^- ^cells (tumors with >10% of CD44^+^/CD24^- ^cancer cells) in 22% of tumor samples. The prevalence of these cells correlated with distant metastasis but no other clinical parameters. Our data suggest that the CD44^+^/CD24^- ^population plays a critical role in the invasive step of metastasis. Thus, distant metastasis in patients with elevated levels of CD44^+^/CD24^- ^cells may be related to enhanced invasiveness of cancer cells. It is possible that the establishment of growth at sites of metastasis is controlled by a distinct set of genes whose expression is unrelated to the CD44^+^/CD24^- ^phenotype. An emerging opinion is that reduced expression of genes involved in cell-cell communication initiates invasion, whereas re-expression of genes involved in cell-cell communication is essential for survival and reattachment of metastases [45]. Therefore, metastatic growth may be primarily determined by signaling pathways that control the re-expression of cell-cell communication genes, which may be further influenced by the organ-specific microenvironment. In this regard, the transforming growth factor-β-activated signaling pathway is suggested to play a significant role in growth of cancer cells at sites of metastasis [13,14]. Although previous studies have identified lung metastasis signature genes using MDA-MB-231 cells as a model system [30], our studies reveal that the same set of lung metastasis signature genes is not involved in metastasis of MDA-MB-468 cells. Thus, additional studies are required to elucidate the mechanisms of tumor cell growth at sites of metastasis.

### CD44^+^/CD24^- ^phenotype may define breast cancers of basal/myoepithelial origin

Molecular profiling studies have classified breast cancers to five types with distinct prognostic significance: luminal type A, luminal type B, ErbB2-positive, normal-like, and basal type [28,29]. Patients with luminal type A tumors have the most favorable prognosis, whereas patients with basal-type tumors have worst prognosis. Breast cancer cell lines have also been classified into five groups – luminal, basal, mesenchymal, ErbB2-positive, and myoepithelial – based on gene expression profiling [25,27]. As per gene expression profiling, basal and mesenchymal cells are similar except for differential expression of 227 genes. Interestingly, all cell lines that contained CD44^+^/CD24^- ^population are in the basal/mesenchymal or the myoepithelial group (Table 1). Thus, stem/progenitor cells for luminal and ErbB2-positive breast cancers, which represent the majority of breast cancers, remain to be identified. In this regard, a 'side population' of cells with high drug efflux capacity has been described as cancer stem cells for breast cancer, lung cancer, and glioblastoma [46,47]. These side population cells have been identified in breast cancer cell lines of luminal type, and these cells overexpress transporter genes ABCG2 (ATP-binding cassette, subfamily G, member 2) and ABCA3 (ATP-binding cassette, subfamily A, member 3) [46]. Cells that express higher levels of CD24 in mouse are defined as luminal epithelial cells, and Lin^-^CD29^hi^CD24^+ ^cells have been defined as mammary stem cells in mouse [48,49]. Note that all luminal cell types contain disproportionately higher levels of CD44^-^/CD24^+ ^cells (Table 1), and this population may contain cancer progenitor cells corresponding to luminal type of tumors. Identification of cancer stem cells specific for luminal cells, which represent about 70% of breast cancers, may allow improved understanding of signaling events that are involved in discrete steps of breast cancer progression, including metastasis.

## Conclusion

In this report we show the relationship between CD44^+^/CD24^- ^phenotype of breast cancer cells and discrete steps of the metastatic cascade. CD44^+^/CD24^- ^phenotype is associated with enhanced invasive properties and elevated expression of genes involved in invasion. One surprising finding is the lack of correlation between CD44^+^/CD24^- ^phenotype and ability to home and proliferate at sites of metastasis. Furthermore, our studies suggest that stem/progenitor cells defined by CD44 and CD24 identify tumorigenic progenitor cells corresponding to basal type.

## Abbreviations

ADAMTS = a disintegrin and metalloproteinase with thrombospondin; CTGF = connective tissue growth factor; CXCR = CXC chemokine receptor; DMEM = Dulbecco's modified eagle medium; ER = estrogen receptor; FCS = fetal calf serum; IL = interleukin; MMP = matrix metalloproteinases; PBS = phosphate-buffered saline; RANK = receptor activator of nuclear factor-κB; RT-PCR = reverse transcriptase polymerase chain reaction; UPA = urokinase plasminogen activator.

## Competing interests

The authors declare that they have no competing interests.

## Authors' contributions

CS performed the analysis of cell lines for stem cell phenotype, Northern analysis, intracardiac injection, and necropsy of animals. HK performed intracardiac injection and trained others in this technique. RF and CHT conducted imaging studies. SM and SB were responsible for histology. PBN conducted Northern analysis and invasion assays. RG was involved in procuring primary tumor samples that were analyzed in parallel with cell lines. HN was involved in designing all experiments, performing flow cytometry, and writing the manuscript.

## Figures and Tables

**Table 1:** Progenitor cell properties (CD44^+^/CD24^-^) of various breast cancer cell lines

Cell line	CD44^+^/CD24^-^	CD44^+^/CD24^+^	CD44^-^/CD24^+^	CD44^-^/CD24^-^	Tumor type^a ^[24,50-52]	Tissue source^b ^[24,50-52]	Cell type classification [25-27]
MDA-MB-231, TMD-231	85 ± 5	2	0	13 ± 5	AC	Pleural effusion	Mesenchymal
TMD-436	72 ± 5	27 ± 5	0	7	AC	Pleural effusion	Myoepithelial
Hs578T	86 ± 5	10 ± 5	0	2 ± 2	CS	Primary	Mesenchymal
SUM1315	97 ± 3	0	0	3 ± 3	IDAC	Metastatic nodule	Basal
HBL-100	37 ± 5	8	2	52 ± 6	Immortal	Milk	Myoepithelial
MDA-MB-468	3 ± 1	90 ± 6	7 ± 3	0	IAC	Pleural effusion	Basal
MCF-7	0	8 ± 3	87 ± 2	6 ± 2	IDAC	Pleural effusion	Luminal
T47-D	0	0	63 ± 3	37 ± 2	IDAC	Pleural effusion	Luminal
ZR-75-1	0	0	64 ± 1	36 ± 1	IDAC	Ascites	Luminal
BT-474	0	0	78 ± 7	22 ± 7	IDC	Primary	Luminal/ErbB2^+^
SK-BR-3	0	0	84 ± 1	16 ± 1	AC	Pleural effusion	Luminal/ErbB2^+^
DU4475	0	0	6 ± 1	94 ± 1	IDC	Cutaneous nodule	ND
MCF-10A^a^	17 ± 4	5 ± 3	20 ± 10	58 ± 20	Immortal	Fibrocyst	Basal

^a^Percentage of MCF10A cell progenitor and other subpopulation was influenced by species and batch of serum in culture media. AC, adenocarcinoma; CS, carcinosarcoma; IDAC, infiltrating ductal adenocarcinoma; IAC, Invasive adenocarcinoma; IDC, Invasive ductal carcinoma; ND, not determined.

**Table 2:** Metastasis pattern of breast cancer cell lines injected into nude mice via intracardiac route

Cell line	Bone metastasis	Gross lung metastasis	Histologic lung metastasis
TMD-231	10/15	0/15	4/4
TMD-436	0/15	4/15	10/15
MDA-MB-468	0/10	5/10	8/8
SUM1315	0/11	0/11	0/11
Hs578T	0/10	0/10	0/10
DU4475	0/8	0/8	0/8
